# Mononuclear cell therapy reverts cuff-induced thrombosis in apolipoprotein E-deficient mice

**DOI:** 10.1186/1476-511X-11-96

**Published:** 2012-07-31

**Authors:** Leandro C F Lima, Marcella L Porto, Bianca P Campagnaro, Clarissa L Tonini, Breno V Nogueira, Thiago MC Pereira, Elisardo C Vasquez, Silvana S Meyrelles

**Affiliations:** 1 Laboratory of Transgenes and Cardiovascular Control, Department Physiological Sciences, Health Sciences Center, Federal University of Espirito Santo, Vitoria, ES, Brazil; 2 Department of Morphology, Health Sciences Center, Federal University of Espirito Santo, Vitoria, Brazil; 3 Federal Institute of Education, Science and Technology, Vila Velha, ES, Brazil; 4 Department of Pharmaceutical Sciences, University of Vila Velha, Vila Velha, ES, Brazil; 5 Emescam College of Health Sciences, Vitoria, ES, Brazil; 6 Department Physiological Sciences, UFES, Av. Marechal Campos 1468, Vitoria, ES 29043-900, Brazil

**Keywords:** ApoE^-/-^, Mononuclear cells, Thrombus, Cuff model

## Abstract

**Background:**

Stem/progenitor cell-based therapy has successfully been used as a novel therapeutic strategy for vascular diseases triggered by endothelial dysfunction. The aim of this study was to investigate the effects of mononuclear cell (MNC) therapy *in situ* on carotid cuff-induced occlusive thrombus in the apolipoprotein E knockout (apoE^-/-^) mouse.

**Methods:**

Spleen-derived MNCs were isolated from green fluorescent protein (GFP)-transgenic mice for cell treatment. A cuff-induced thrombus model was produced by placing a nonconstrictive silastic collar around the left common carotid artery in 20-week-old female apoE^-/-^ mice. After 10 days, the cuff was removed, and the animals received *in situ* MNCs (Cuff-MNC) or vehicle (Cuff-Vehicle) and were compared with sham-operated animals (Sham).

**Results:**

The histological analysis showed that the MNC treatment reverted occlusive thrombus formation compared to the vehicle and the vessel lumen area to that observed in the Sham group (MNC, 50 ± 4; Vehicle, 20 ± 4; Sham, 55 ± 2 x10^3^ μm^2^; p < 0.01). The animals that underwent the carotid cuff placement developed compensatory vessel enlargement, which was reduced by the MNC therapy. In addition, the treatment was able to reduce superoxide anion production, which likely contributed to the reduced apoptosis that was observed. Lastly, the immunofluorescence analysis revealed the presence of endothelial progenitor cells (EPCs) in the carotid endothelia of the apoE^-/-^ mice.

**Conclusion:**

*In situ* short-term MNC therapy was able to revert cuff-induced occlusive thrombi in the carotid arteries of apoE^-/-^ mice, possibly through the homing of EPCs, reduction of oxidative stress and decreased apoptosis.

## Background

Endothelial dysfunction is widely accepted as a fundamental pathophysiological mechanism linking various cardiovascular risk factors, such as atherosclerosis and thrombosis [1,2]. Despite progress in drug treatments, researchers have been challenged to develop new successful approaches to repair these injuries. In particular, emphasis has been placed on the experimental and clinical potential of endothelial progenitor cells (EPCs) to promote vascular repair and the reversal of ischemic injury in various tissues [3,4].

The development of ischemic diseases in conditions of hypercholesterolemia is aggravated by endothelial dysfunction [5,6]. Therefore, the apolipoprotein E-deficient (apoE^-/-^) mouse is considered to be the most relevant model because the animals develop spontaneous hypercholesterolemia and consequently endothelial dysfunction accompanied by arterial lesions that are similar to those observed in humans [1,7]. Moreover, recent studies have demonstrated that the female gender is an important factor in the development of endothelial dysfunction and atherosclerosis [1,8,9]. Because the integrity of the endothelium is important to suppress thrombosis, the cuff placement model may be an interesting strategy to study thrombogenesis by the impairment of arterial distensibility.

Recently, we reported that the intravenous administration of mononuclear cells (MNCs) attenuates the progression of atherosclerosis in the aortas of apoE^-/-^ mice by mechanisms that include the homing of EPCs, a decrease in oxidative stress and an upregulation of endothelial nitric oxide synthase expression [10]. In the present study, we tested the hypothesis that *in situ* MNC therapy could revert cuff-induced thrombosis in apoE^-/-^ mice.

## Materials and methods

### Animals

ApoE^-/-^ female mice (20 weeks old) were randomly divided into three groups: an apoE^-/-^ control group (Sham, n = 8), apoE^-/-^ treated *in situ* with MNC group (Cuff-MNC, n = 8) and apoE^-/-^ treated with DMEM group (Cuff-Vehicle, n = 8). The animals were obtained from the animal facilities of the Federal University of Espirito Santo. The mice were fed a normal chow diet and water *ad libitum* and were housed individually in temperature-controlled rooms (22°C) under a 12 h light/dark cycle. All of the procedures were conducted in accordance with the institutional guidelines for animal research, and the protocols were previously approved by the Institutional Ethics Committee for Use of Animals (CEUA 007/2008).

### Carotid cuff placement

**Figure 1:**
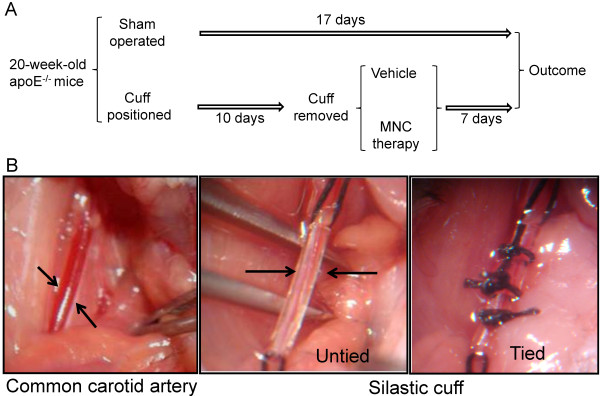
**Scheme of the study and carotid artery cuff positioning.** Panel **A** shows the time course of the procedures and treatment of the animals. Panel **B** presents macroscopic views of an opened neck of an apoE^-/-^ mouse showing, from left to right, a dissected left common carotid artery, a silastic cuff being placed around the artery and the cuff tied around the artery.

The apoE^-/-^ mice (n = 24) were anesthetized with ketamine/xylazine (91.0/9.1 mg/kg, IP). The animals first underwent splenectomy: (a) the spleen was dissected through a lateral incision of the abdomen, (b) the vessels were carefully ligated using a 6.0 silk, and, (c) after the removal of the spleen, the abdomen was closed layer by layer with a 6.0 silk. Immediately afterward, the carotid artery injury was performed based on a previously described method [11,12]. Cuffs were produced using a silastic tubing (length, 4 mm; internal diameter, 0.3 mm; external diameter, 0.5 mm, Clay-Adams, Parasippany, NJ, USA) and stored at 70% ethanol. As illustrated in Figure 1, access to the common carotid artery was through a sagittal anterior neck incision. The common carotid artery was dissected free from the surrounding connective tissue, avoiding damage to the vagus nerves and the carotid bodies. The nonconstrictive cuff was placed around the left common carotid artery, and the axial edges of the cuff were approximated by the placement of 3 circumferential silk ties (Cuff groups; n = 16). In the sham-operated mice (Sham group; n = 8), the neck wound was closed in one layer with interrupted 6.0 silk sutures. The entry wound was closed, and the animals were returned to their cages for recovery from the anesthesia. An inside diameter of 0.3 mm was constrictive and resulted in stenosis of the common carotid artery.

### MNC isolation and treatment

Green fluorescent protein (GFP)-transgenic mice were euthanized with a sodium thiopental overdose (100 mg/kg, i.p.). The spleens were explanted and mechanically minced, and the tissue homogenates were placed in 1 mL of Dulbecco’s Modified Eagle Medium (DMEM, Sigma-Aldrich, St. Louis, MO, USA) at 4°C; the MNCs were isolated by gradient centrifugation using Histopaque 1083 solution (Sigma-Aldrich) and resuspended in DMEM. The MNCs were counted using a Neubauer chamber for the further *in situ* treatment.

After 10 days of carotid artery stenosis induction, under anesthesia, the cuff was removed, and the MNCs (10^6^ cells, resuspended in 20 μL of DMEM) were topically applied once *in situ* where the cuff was positioned in 50% of the animals (MNC group; n = 7). The cuff was removed in the other 50% of the animals, and they received vehicle (20 μL DMEM) only (Vehicle group; n = 8). The apoE^-/-^ animals that were sham-operated did not receive any treatment and were used as the hypercholesterolemic control (Sham group; n = 8). The donor-derived cells were identified using GFP expression (green cells), which has been used as a reporter gene [13], with a B-2E/C Nikon filter that provided GFP excitation and emission at 471 and 504 nm, respectively. Figure 1 shows a schematic illustration of the time-course of the cuff-induced artery stenosis and cell therapy.

### Atherosclerotic lipid deposition quantification and morphometry

A week after the treatment with the MNCs, the animals were euthanized with a sodium thiopental overdose (100 mg/kg, i.p.) and perfused via the left ventricle with Krebs-HEPES for 10 min at 100 mm Hg constant pressure. The left common carotid artery was removed and embedded in OCT compound (Tissue-Tek; Sakura Finetek USA, Torrance, CA, USA). The samples were stored at -80°C until further use. Transverse (8 μm) cryosections (Leica, CM 1850, Leica, Wetzlar, Germany) were prepared and mounted on parallel series of gelatin-coated slides and stained with Oil-Red-O (Sigma-Aldrich) to detect neutral lipids and with hematoxylin-eosin (HE) for the conventional histopathology analysis and morphometry.

The images of the mid-section of the carotid artery were captured with a color video camera (VKC 15 0, Hitachi, Tokyo, Japan) connected to a microscope (Olympus AX70, Olympus, Center Valley, PA, USA) and were analyzed using Image J program (National Institute of Health). An examiner blinded to the experimental groups performed the image analysis to prevent any bias in the interpretation of the results. By using a 20x objective, the vessel cross-sectional area (V_CSA_), which was bordered by the external elastic membrane, the lumen cross-sectional area (L_CSA_), and the arterial wall (intima-media) area (V_CSA_-L_CSA_) were calculated. The vascular remodeling ratio was obtained by dividing each animal’s V_CSA_ (apoE^-/-^ Vehicle- and apoE^-/-^ MNC-treated groups) by the average V_CSA_ of the apoE^-/-^ Sham group, and each sample was scored for the absence of remodeling (0.95-1.05), inward remodeling (< 0.95), or outward remodeling (> 1.05) [14].

### Immunofluorescence

Immunofluorescence was performed according to standard protocols for EPC detection. Cross-sections (8 μm) were cut on a cryostat and placed on gelatin-coated slides. The sections were air-dried, and the slides were fixed for 5 min in acetone at -20°C. The slides were incubated with primary antibodies for 2 hours at room temperature conjugated with fluorescein isothiocyanate (FITC) against the stem cell growth factor receptor (CD117), 1:150 (BD Pharmingen, San Diego, CA, USA) and labeled with phycoerythrin (PE) against stem cell antigen-1 (Sca1), 1:150 (BD Pharmingen).

Typical images were captured with a color video camera (Nikon Digital Sight DS - U2, Nikon Instruments Inc., Melville, NY, USA) connected to a fluorescence microscope (Nikon Eclipse Ti-S). The green fluorescence (FITC) was collected through a B-2E/C Nikon filter that provided FITC excitation and emission at 490 and 525 nm, respectively; the red fluorescence (PE) was collected through a G-2E/C Nikon filter that provided PE excitation and emission at 545 and 576 nm, respectively.

To ensure that the fluorescence observed was only due to antibody labeling, autofluorescence background was eliminated using samples before incubation with antibodies as negative controls, as follows: (a) tissue sections were visualized using appropriate filters for each fluorochrome used in the protocol (PE and FITC); (b) gain and exposure parameters were adjusted to cancel the autofluorescence of each fluorochrome separately, and (c) the samples were incubated with the fluorochrome-conjugated antibodies (Sca-1-PE and CD117-FITC) and photographed using the same gain and exposure values established for the negative control.

### TUNEL assay

Apoptotic cells were detected with TUNEL assay using the Cell Death Detection Kit (POD, Roche, Mannheim, Germany) according to the specifications and instructions recommended by the manufacturer. Briefly, after washing in PBS, the sections were incubated with 3% H_2_O_2_ in methanol for 10 minutes. For detecting the apoptotic DNA fragments in the cuff-induced vascular remodeling cells, 50 μL of the TUNEL reaction mixture was added and incubated for 1 hour in a light-protected humidified chamber at room temperature. Typical images were captured with a color video camera (Nikon) connected to a fluorescence microscope (Nikon) using a B-2E/C Nikon filter that provided FITC excitation at 490 nm; the emission was monitored at 525 nm.

### Dihydroethidium fluorescence of carotid arteries

The oxidative fluorescent dye dihydroethidium (DHE) was used as in previous study [10]. The unfixed frozen left common carotid arteries were cut into 8-μm-thick sections and placed on glass slides. The samples were incubated with DHE (2x10^-6^ mol/L) in a light-protected humidified chamber at 37°C for 30 minutes. The tissue sections were imaged using an inverted fluorescence microscope (Nikon Ti-S) with a G-2E/C Nikon filter that provided excitation at 530 nm and emission at 610 nm.

### Cholesterol levels

A blood sample (200 μL) was collected from the carotid artery of each animal, and the plasma total cholesterol was measured using a commercial colorimetric kit (Bioclin, Belo Horizonte, Brazil).

### Statistical analyses

The data are presented as the mean ± SEM. The normality of the variables was evaluated using the Kolmogorov-Smirnov test. The statistical analysis was performed using one-way ANOVA, followed by the Tukey *post hoc* test for multiple comparisons. The Student’s *t* test for independent samples was used when appropriate. The statistical significance was set at p < 0.05.

## Results

The plasma total cholesterol levels of the 22-week-old animals fed a regular chow diet were similar among the apoE^-/-^ Sham group and those that underwent the cuff-induced artery stenosis, followed by the topical administration of vehicle or cell therapy with MNC (372 ± 48, 369 ± 33 and 350 ± 33 mg/dL, respectively, p > 0.05). The body weight did not differ among the three groups of apoE^-/-^ mice at the end of the experiment.

**Figure 2:**
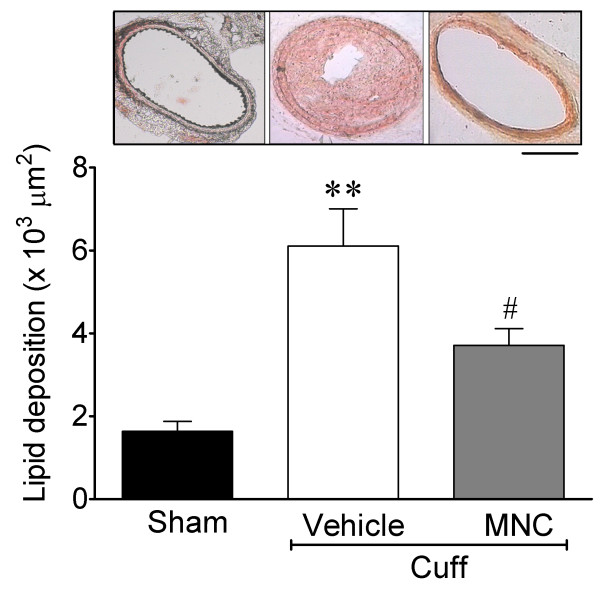
**Effects of cuff-induced stenosis on the lesion area in the common carotid artery and the effect of MNC therapy in apoE**^**-/-**^**mice.** The top panel shows typical histological micrographs stained with Oil-Red-O comparing an artery with stenosis that received vehicle, an artery with stenosis treated with mononuclear cells (MNCs) and an artery from a Sham apoE^-/-^ mouse. The bar graphs show the average lesion areas of the three groups (n = 8 per group) of apoE^-/-^ mice. The values are the means ± SEM. **p < 0.01 *vs* Sham; ^#^p < 0.05 *vs* Vehicle (ANOVA). Scale bar = 100 μm.

Figure 2 shows the effect of the MNC therapy on the cuff-induced carotid lesion area. As observed in the typical carotid cross-sections, this procedure resulted in a large lesion area at the site where the cuff was positioned for 10 days, when compared with the apoE^-/-^ Sham mouse. The animals treated with the MNCs showed a marked reduction of occlusive thrombi. As summarized in Figure 2 (bar graph), the positioning of the silastic cuff around the common carotid artery for 10 days, its removal and the topical application of vehicle at this site (Cuff-Vehicle group) resulted in a significant lipid deposition area when compared to the sham-operated animals (Sham group) (6.11 ± 0.89 *vs* 1.64 ± 0.24 x10^3^ μm^2^, p < 0.01). After 7 days, the MNC therapy (Cuff-MNC group) caused a significant reduction in the vascular lipid deposition area (3.71 ± 0.40 x10^3^ μm^2^, p < 0.01), but the area was still significantly (p < 0.05) larger than in the Sham group.

**Figure 3:**
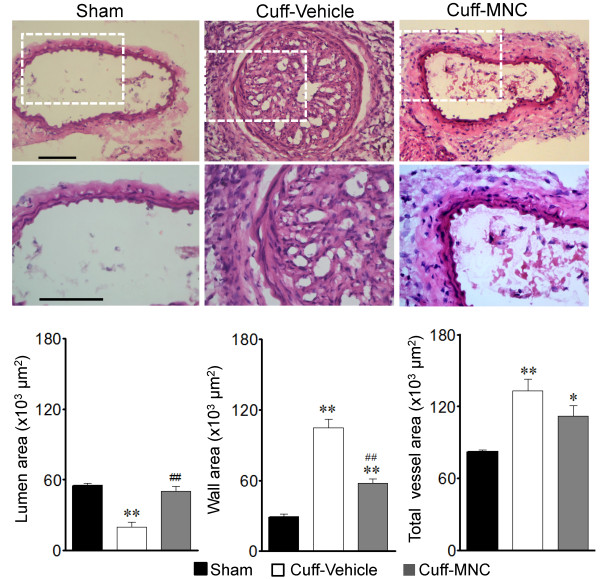
**Effect of mononuclear cell (MNC) therapy on cuff-induced stenosis in the common carotid arteries of apoE**^**-/-**^**mice.** Tope panel microphotographs are typical cross-sections of common carotid arteries of apoE^-/-^ mice that underwent sham-operation (Sham) or cuff-induced stenosis followed by the treatment with MNCs or vehicle. Scale bar = 100 μm. Bottom bar graphs show the data of the effects of MNC therapy on the lumen area, wall area and cross-sectional area. The values are means ± SEM. *p < 0.05 and **p < 0.01 compared to Sham group (ANOVA); ^#^p < 0.05 and ^##^p < 0.01 compared to Vehicle group (ANOVA) (n = 8 per group).

The second outcome we analyzed was the effect of the common carotid artery positioning of the cuff on the morphological parameters. The effect of this procedure is observed in the typical carotid cross-sections shown in Figure 3 (top panels): in the apoE^-/-^ mouse that underwent cuff positioning (Cuff-Vehicle), an increased wall thickness, a highly vascularized thrombus occupying most of the lumen and an increased cross-sectional area when compared with the apoE^-/-^ Sham mouse is observed, whereas the apoE^-/-^ mouse that received the topical application of MNCs on the injured vessel after the cuff removal show a marked reduction of these parameters. Figure 3 (bottom panels) summarizes the effect of the cuff positioning and the cell therapy on the indicated parameters. The vascular lumen area was significantly reduced in the Cuff-Vehicle group compared to the Sham group (20 ± 4 *vs* 55 ± 2 x10^3^ μm^2^, p < 0.01), a result that was completely reversed after 7 days of treatment with MNCs (50 ± 4 x10^3^ μm^2^, p < 0.01). In addition, the vessel wall area was significantly thickened in the Cuff-Vehicle group compared with the Sham group (105 ± 8 *vs* 29 ± 3 x10^3^ μm^2^, p < 0.01), and this was significantly reduced by the MNC therapy (58 ± 4 x10^3^ μm^2^, p < 0.01). The total cross-sectional vessel area was significantly larger in the Cuff-Vehicle group compared with the Sham group (133 ± 9 *vs* 82 ± 2 x10^3^ μm^2^, p < 0.01) and was partially reduced by the MNC therapy (112 ± 9 x10^3^ μm^2^, p < 0.05). As a result of the latter parameter, the calculated remodeling index showed the occurrence of an outward remodeling in the Cuff-Vehicle group and a significant reduction of this index by the MNC therapy (1.61 ± 0.09 *vs* 1.35 ± 0.06, p < 0.05), with the Sham group as the reference value.

**Figure 4:**
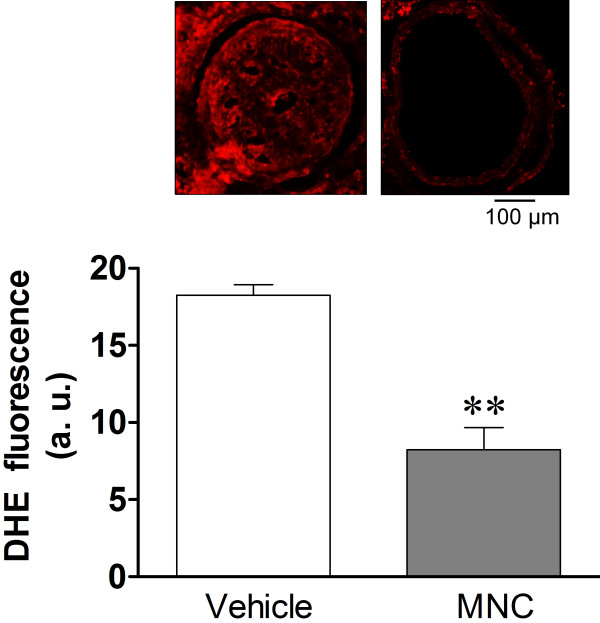
**Superoxide anion production in the lesion area of common carotid arteries with cuff-induced stenosis and the beneficial effect of mononuclear cell (MNC) therapy in apoE**^**-/-**^**mice.** The top panel presents representative histological micrographs stained with dihydroethidium (DHE), showing intense bright ethidium fluorescence (red) in the Cuff-Vehicle compared to the Cuff-MNC mouse. The bar graph shows the average DHE fluorescence (AU: arbitrary units) comparing the Cuff–Vehicle to Cuff-MNC mice (n = 8 per group). The values are the means ± SEM. **p < 0.01 compared to Cuff-Vehicle (Student’s *t* test). Scale bar: 100 μm.

**Figure 5:**
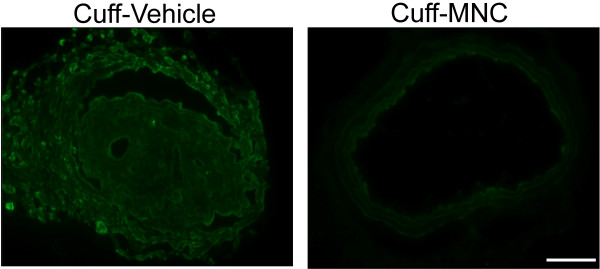
**Representative images of the results of the TUNEL assay showing that the mononuclear cell (MNC) therapy markedly reduces the process of apoptosis (green fluorescent cells) in the common carotid artery under stenosis (Cuff) compared with the Vehicle apoE**^**-/-**^**mouse.** Scale bar: 100 μm.

Third, we investigated the effect of the carotid artery cuff-induced stenosis and its treatment with cell therapy on both oxidative stress and apoptosis. As shown in a typical microphotograph in Figure 4 (top panel), the dihydroethidium oxidative assay of the carotid artery cross sections of the apoE^-/-^ mice that were subjected to cuff-induced stenosis reveal intense ethidium fluorescence (indicative of superoxide production) in the untreated mouse (Vehicle) but not in the mouse treated with MNCs. On average, the carotid arteries from the apoE^-/-^ mice that underwent the cuff-induced stenosis showed approximately 50% less (p < 0.01) ethidium fluorescence in the MCN group than in the Vehicle group (Figure 4, bottom panel). Similarly, the results of the TUNEL assay show an intense fluorescence (indicative of apoptosis) in a cuff-induced injury in the carotid artery of an untreated apoE^-/-^ mouse but not in the vessel of the mouse subjected to the cell therapy (Figure 5).

**Figure 6:**
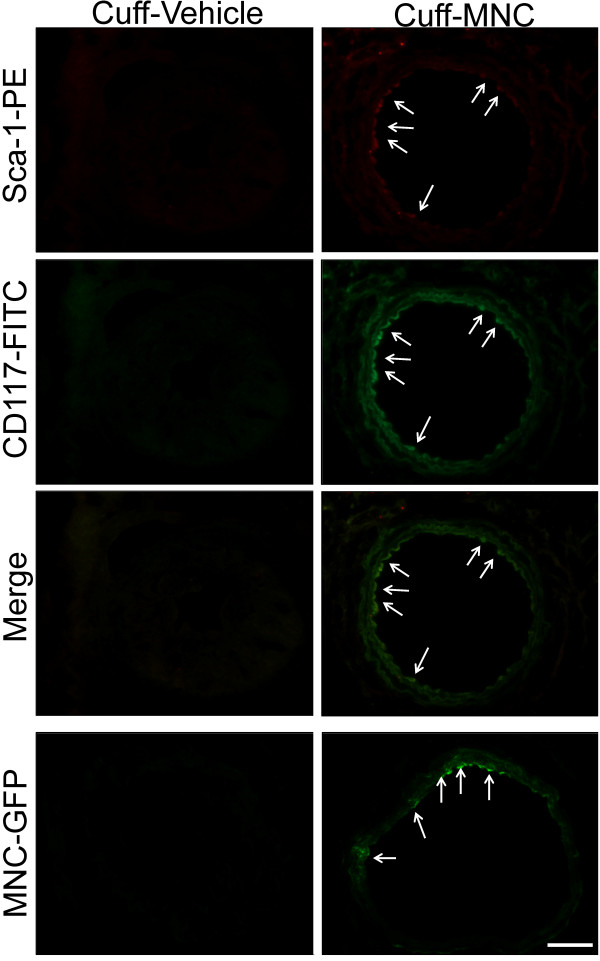
**Localization of endothelial progenitor cells (EPC) and green fluorescent protein (GFP)-derived mononuclear cells (MNC).** The photomicrographs are typical common carotid cross-sections stained for the markers Sca-1-PE (stem cell antigen 1-phycoerythrin) and CD117-FITC (stem cell growth factor receptor-fluorescein isothiocyanate), showing an intense immunofluorescent reaction in MNC-treated apoE^-/-^ animals (indicated by the arrows and merged image) in contrast with the lack of fluorescence in the Cuff-Vehicle mice. The self-fluorescence of the MNC-derived GFP confirms the presence of the MNCs in the endothelial vessel of the treated (but not the untreated) mouse and indicates that the treatment was able to recruit endogenous EPCs. Scale bar: 100 μm.

Another outcome of the cuff-induced stenosis in the apoE^-/-^ mice was the homing of EPCs to the injured lesion (indicative of vessel injury repair). Figure 6 shows a typical carotid artery cross-section, revealing specific fluorescence signals in the mice treated with MNC but not in the animals treated with vehicle. The endogenous fluorescence of the MNC-derived GFP confirmed the presence of these cells in the carotid artery of MNC-treated mice but not in the vehicle-treated mice (Figure 6, bottom panel). The homing of EPCs was confirmed by the detection of immunofluorescence of Sca-1-PE and CD117-FITC, markers of EPCs, showing intense Sca-1 and CD117 expression (as shown in merged images) in the carotid endothelium of MNC-treated apoE^-/-^ mice in comparison with untreated mice.

## Discussion

In the present study, we observed that the *in situ* short-term MNC therapy reverted cuff-induced occlusive thrombi in the carotid arteries from apoE^-/-^ mice by the homing of EPCs, the reduction of ^·^O_2_^−^ production and decreased apoptosis, even under conditions of hypercholesterolemia.

Perivascular cuff placement has been used to induce disturbed flow, accelerating endothelial dysfunction and atherosclerotic lesions in medium- and large-sized arteries in both rabbits [15] and mice [11,16,17]. Sasaki et al. [18] adapted this model to a simultaneous ligation of the carotid artery for 7 days to induce intra-cuff thrombosis in normocholesterolemic young mice. By a simple perivascular cuff placement in our study, we were able to generate occlusive thrombus formation in the adult apoE^-/-^ mice in a short time. Our achievement can be explained by the concurrence of hypercholesterolemia, endothelial dysfunction, age (20 weeks old) and gender, i.e., female apoE^-/-^ mice may be more susceptible to develop endothelial dysfunction [1,8,9], and a female gender may facilitate the progression of vessel stenosis. We expect that this thrombus formation model could be utilized in the assessment of various known and yet-unknown antithrombotic agents in further investigations.

In this regard, cell therapy has newly emerged for the treatment of cardiovascular diseases. Stem/progenitor cells, a subpopulation of MNCs, have unique characteristics that make them important for therapeutic purposes: they are undifferentiated and unspecialized and can divide symmetrically and asymmetrically for long periods of time [19-21]. As a consequence of the recent advances in stem/progenitor cell biology, these cells have been used to repair endogenous vascular lesions [10,22], and our study contributes to this objective, as the MNCs have antithrombotic properties in apoE^-/-^ mice, even without additional drug treatment. More specifically, it is known that EPCs are a subset of bone marrow-derived cells committed to the maintenance and preservation of vascular turnover, remodeling and homeostasis [23]. EPCs are immature cells that have the capacity to be mobilized from the bone marrow into the bloodstream in response to growth factors and cytokine release [24], may differentiate into endothelial cells and participate in the re-endothelialization of injured vessels and neovascularization of ischemic lesions, suggesting that EPCs play major roles in the pathogenesis of cardiovascular diseases [25].

The present study provides data on the parameters of vascular remodeling in the carotid artery cuff-induced thrombosis model of hypercholesterolemic animals. The main findings are that both groups, Cuff-MNC and Cuff-Vehicle, developed carotid compensatory enlargement compared to the Sham animals, however the MNC therapy was able to significantly reduce this enlargement. Vascular remodeling is an adaptive process that occurs as a response to changes in hemodynamic conditions [26,27], and we speculate that the outward remodeling was reduced by the MNC therapy as a direct consequence of thrombus reversion or dissolution. Our hypothesis is that the EPCs from the MNC fractions are involved in the antithrombotic effect, as we observed the vascular homing of the EPCs in the carotid arteries of the apoE^-/-^ mice treated with MNC *in situ*. As shown in Figure 6, the immunofluorescence analysis showed the presence of Sca-1 and CD117, both markers for EPCs [28,29] and GFP-positive cells, in the carotid arteries of the apoE^-/-^ mice. These findings indicate the presence of donor EPCs in the thrombotic area, suggesting that the therapeutic effect of the MNCs was at the level of the arterial wall in those animals. Our experimental model of the nonconstrictive cuff may involve an acute turbulent flow and the consequent exposition of the subendothelium [18], which could be an effective mechanism of the mobilization and homing of EPCs at the damaged area [30]. Although our administration route was extravascular, other investigators demonstrated the ability of progenitor cells to migrate from the adventitia into the intima [31], corroborating our results. Moreover, others have established that there is a protective role of the progenitor cells that could become “exhausted” with aging and the prolonged exposure to risk factors, such as hypercholesterolemia [32]. Compared to autologous transplantation, the use of healthy GFP mice, which were used as the donors of the MNCs, provided feasible ways of detecting the localization of the cells and their effectiveness in cell treatment.

Disorders such as accelerated vascular damage and impaired EPC function are related to increased oxidative stress, which affects the mobilization, function and survival of EPCs [33]. Consequently, interventions that are able to potentiate tissue antioxidant power, such as pharmacological and cell therapies, have been proven to benefit vascular integrity [34-36]. Thus, we investigated the possible mechanisms by which EPCs could locally mediate the attenuation of thrombosis in apoE^-/-^ mice. First, we tested the hypothesis that the beneficial effects of MNC therapy could include the reduction of oxidative stress and apoptosis. Our data show that the carotid arteries of apoE^-/-^ mice treated with MNCs exhibited a marked decrease in the production of ^·^O_2_^−^ and a significant reduction in apoptosis; these results supports the idea that the promotion of lowered oxidative stress and the augmentation of cell viability is another important mechanism by which MNC therapy attenuates thrombosis. In contrast, the cuff-induced thrombus model exhibits greater endothelial dysfunction and ROS production, stimulating apoptotic and inflammatory gene expression and inducing chronic injury.

Our data suggest that cell therapy could change the thrombotic microenvironment and provide molecular and cellular mechanisms for the organization and recanalization of thrombi by either autocrine or paracrine means [29]. Platelet adhesion under conditions of high shear stress, which occurs in arteries with endothelial dysfunction, is not only the initial step of arterial thrombosis but also an extension and perpetuation of thrombus formation [37]. Thus, even though EPCs are involved in the synthesis and release of soluble agonists that enhance platelet aggregation (e.g., 5-HETE, LTB4), the antithrombotic properties of EPCs may be mediated by an autocrine/paracrine regulation of platelet function. Our findings show that cell therapy reduced ^·^O_2_^−^ production and a concomitant decrease in endothelial TUNEL positivity, suggesting a reparative effect of EPCs and improvements in endothelial function, re-endothelization and neovascularization. As previously demonstrated by Porto et al. [10], the increased number of EPCs provided by MNC treatment of apoE^-/-^ mice leads to decrease in ROS production and an increase in NO bioavailability. This free radical is a powerful inhibitor of platelet aggregation that works by stimulating an increase in the intracellular levels of cyclic guanosine monophosphate (cGMP) and reduces thromboxane A2 synthesis [38]. Moreover, a reduced stimulation of cell proliferation could be explained by apoptotic mechanisms. Therefore, it seems reasonable to speculate that the reduced apoptosis that we observed upon MNC treatment in the present study promotes the inhibition of phosphatidylserine expression [39,40] and platelet procoagulant function. Based on other findings [41], procoagulant platelets may also contribute to the inflammatory response by generating proinflammatory factors and by enhancing leukocyte adhesion. Our hypothesis is that EPC acted to recognize the thrombi by two major processes: proteolytic activity (which causes the shedding of platelet receptors) and EPC homing (by autocrine modulation), which increases the speed of clot organization and reperfusion, recanalization and consequently the dissolution of the thrombus, minimizing the time of that downstream tissues are exposed to ischemia [42]. Other important factors that could contribute to thrombus dissolution include arterial blood flow and pressure, which are expected to accelerate the recanalization [42]. Finally, efforts to restore the normal vascular redox balance and reduction of apoptosis by MNC therapy may provide a therapeutic avenue for reducing platelet-dependent thrombi and accelerating their dissolution.

However, our study has some limitations that need to be addressed. First, it would be interesting to demonstrate the direct relationship between cuff placement and hemodynamic alterations. The measurement of hemodynamic parameters, such as blood flow velocity or computational fluid dynamics, would help to better explain our findings. Second, a careful time course study could clarify the progression of endothelial cell damage in the neointima that is accompanied by thrombus without plaque disruption. This could confirm whether thrombus formation occurs independently of atherosclerosis because luminal thrombus rarely occurs in mice [43]. In this study, we used a GFP-transgenic mouse as an MNC donor because it allowed us to identify the transplanted cells, but this animal is normolipidemic; in contrast, autologous transplantation is usually used in clinical practice. Further investigations should compare the efficacy of antithrombotic MNC from apoE^-/-^ mice versus MNC from (GFP)-apoE^+/+^ donors.

In conclusion, we have shown that *in situ* MNC therapy inhibited occlusive thrombus formation in the carotid arteries of apoE^-/-^ mice. Our data provide evidence that the protective mechanisms of MNC therapy include the homing of EPCs and the reduction of oxidative stress and apoptosis. The present data provide important evidence that regenerative cell-based therapy has the potential to become an effective adjuvant in the treatment of patients with dyslipidemia and thrombosis.

## Competing interests

The authors declare that they have no competing interests.

## Authors’ contributions

LCF and MLP performed the experimental analysis and acquisition of data, analysis and interpretation of the data. TMCP performed the statistical analysis and helped to draft the manuscript. BVN contributed to the histology analysis. CLT and BPC participated in the study’s design. SSM and ECV contributed to the conception, design and supervision of the study and interpretation of data. All authors read and approved the final version of the manuscript.
